# Oral absorption of high molecular weight polyethylene glycols: mechanistic, safety and regulatory insights

**DOI:** 10.3389/fdsfr.2026.1851318

**Published:** 2026-08-28

**Authors:** Chiara G. M. Gennari, Sara Azari, Antonella Casiraghi, Umberto M. Musazzi

**Affiliations:** Department of Pharmaceutical Sciences, University of Milan, Milan, Italy

**Keywords:** gastrointestinal absorption, MDR rule 21 classification, medical devices, osmotic laxatives, polyethylene glycols (PEG 3350/4000), safety assessment of high-MW PEGs

## Abstract

The gastrointestinal absorption of macromolecules represents a major challenge in both drug development and regulatory evaluation, as the intestinal barrier is highly effective in limiting the systemic uptake of large molecules. Nevertheless, understanding the mechanisms that govern macromolecule permeability is essential for assessing the safety, efficacy profile, and regulatory qualification of substance-based healthcare products. Polyethylene glycols (PEGs) represent a useful reference system for understanding the physiological limits of macromolecule absorption and for supporting evidence-based regulatory decision-making. Indeed, PEGs are widely used as excipients and active ingredients in numerous healthcare products due to their chemical stability, low immunogenicity, and good tolerability; however, their therapeutic use is strongly influenced by their gastrointestinal absorption. Low-molecular-weight (lMW) PEGs (200–600 g/mol) can be absorbed through the gastrointestinal mucosa and therefore are effective probes for testing gastrointestinal functionality. On the contrary, high-MW (hMW) PEGs (e.g., polyethylene glycol 3350 and PEG 4000), are negligible absorbed, acting as osmotic laxatives. Under Regulation (EU) 2017/745, hMW PEGs are classified as Class IIb substance-based medical devices. This review critically analyzes the current evidence on the gastrointestinal fate of PEGs with different molecular weights and discusses their relevance for the interpretation of macromolecule absorption. The overall evidence is particularly relevant for the regulatory qualification of PEG-containing healthcare products.

## 1 Introduction

Gastrointestinal absorption of macromolecules (high molecular weight molecules, including proteins, peptides and polymers) is a central topic in digestive physiology and pharmacokinetics, as it highlights the intestine’s extraordinary ability to merge two seemingly opposing functions: promoting the selective passage of nutrients while simultaneously preventing the entry of potentially harmful substances. The intestinal barrier, composed of specialized enterocytes, tight junctions that regulate paracellular permeability, a thick layer of mucus, and a complex mucosal immune system, is designed to drastically limit the transit of large molecules. Under physiological conditions, in fact, most macromolecules are degraded before absorption or almost excluded from passage through the epithelium. Gastrointestinal absorption of macromolecules is limited by their inability to cross intestinal barriers and susceptibility to the degradative environment.

The general reasons for poor gastrointestinal absorption of macromolecules are related to their size and permeability properties, enzymatic digestion and acid environment. Macromolecules are too large to easily pass through the tight junctions between intestinal epithelial cells (paracellular pathway), and their hydrophilic nature makes it difficult to pass through cell membranes (transcellular pathway). Moreover, the gastrointestinal tract is rich in proteases and peptidases that break down complex macromolecules before they can be absorbed. The varying pH along the gastrointestinal tract can degrade macromolecules. Only in specific circumstances, such as in newborns or in the presence of inflammation and mucosal damage, endocytosis or an increase in paracellular permeability can occur, allowing the transit of larger compounds.

The in-depth understanding of pharmacokinetics of macromolecules raises particular interest in the scientific and regulatory community since many of them are parts of medicinal products or medical devices. Having a clear view not only of the mechanism of action of a macromolecule, but also of their absorption pathways is nowadays crucial for a proper regulatory qualification of the products. This is critical for macromolecules (e.g., fructooligosaccharides, xyloglucan, glucomannan, polyethylene glycol) that can be qualified as substance-based medical devices in agreement with Rule 21, Annex VIII of Regulation 745/2017 on medical devices (European Union, 2017; MDCG, 2021). The higher the risk of systemic biodistribution, the higher the class of medical devices.

In this context, polyethylene glycol (PEG) represents a particularly useful experimental model for discussing regulatory and scientific challenges in properly qualify substances that may be used in clinics for not-pharmacological purposes. PEGs, also known as macrogol, are widely used as excipients or finished products in many healthcare products, such as medicinal products, medical devices, cosmetics and food supplements. A family of polyether compounds produced by polymerization of ethylene oxide with water or ethylene glycol. According to their molecular weight, PEGs are classified in low MW (200–600 g/mol) (clear, viscous liquids) and high MW (≥4,000 g/mol) (waxy solids or powders). PEGs exert primarily physical effects, by coordinating water molecules, and, therefore, they are used as osmotic laxatives (Schiller et al., 1988). Moreover, their chemical neutrality and stability make it an ideal tracer for assessing intestinal permeability, since its behaviour in the lumen and its possible absorption depend almost exclusively on molecular size (Ma et al., 1990). By studying PEGs of different masses, it is possible to observe how absorption progressively decreases as the molecular weight increases: the lightest forms can cross the barrier in minimal quantities, while intermediate forms show negligible absorption, and high-molecular-weight forms are known by regulatory authorities as non-permeable molecules (EMA, 2020). This inverse relationship between size and permeability allows to precisely define the “cut-off size” of the intestinal epithelium, offering valuable information for both basic research and clinical applications.

The aim of our review is to discuss the effects and toxicological implications of osmotic laxatives based on high molecular weight (hMW) PEGs, based on current knowledge, and the regulatory framework for these medical devices.

For this review, literature was identified through searches in PubMed, Scopus and Google Scholar using combinations of the following terms: “polyethylene glycol”, “PEG 400”, “PEG 3350”, “PEG 4000”, “absorption”, “bioavailability”, “impurities”, “intestinal permeability”, and “osmotic laxatives”. Studies were selected based on mechanistic relevance, analytical robustness, and regulatory significance. Given the heterogeneity and limited number of pharmacokinetic investigations on hMW PEGs, a formal systematic methodology was not considered appropriate for the aims of this manuscript.

## 2 Use and mechanism of action

PEGs are synthetic polymers that are not degraded or absorbed during the transit in the gastrointestinal tract and are excreted intact. PEGs are well-known pharmaceutical excipients used in many oral dosage forms, but they are also used in clinics for different therapeutic indications, based on the molecular weight (MW) of their polymeric chains.

On the one side, PEG 400 is used in clinic, for example, in intestinal permeability tests, to evaluate barrier alterations in pathological conditions, such as celiac disease or chronic inflammatory bowel disease (Ma et al., 1990). Indeed, as inert and not-metabolized macromolecule, it can be detected unchanged in the urines of patients (Kuki et al., 2022), becoming a powerful tool for understanding the mechanisms that regulate intestinal absorption and for exploring the physiological restrictions of the mucosal barrier.

On the other side, hMW PEGs are present in medicinal products and medical devices as an osmotic agent intended to be used for treating constipation and/or for cleaning the gastrointestinal tracts before other diagnostic investigations. PEG 3350 and PEG 4000 formulations are class IIb medical devices, intended for short-term use (up to 30 days), according to Annex VIII of Regulation 2017/745/EU, classification rule 21 (MDCG, 2021). PEG 3350 and PEG 4000 formulations belong to a group of laxatives called osmotic laxatives and they are intended for the treatment of chronic and occasional constipation and fecaloma. They are available as over-the-counter powders for constipation and prescription-strength or electrolyte-balanced solutions for bowel prep. They come in sachets, in the form of powder, which are mixed with water. The laxative activity of hMW PEG is related to its capacity to retain relevant amounts of water in the intestinal lumen, which makes the stool softer and easier to expel. Indeed, previous studies have demonstrated that PEG shows a strong interaction of PEG with adjacent water molecules (Kjellander and Florin, 1981; Breen et al., 1988; Antonsen and Hoffman, 1992). In addition, the increase of endoluminal pressure due to the enrichment of the fecal mass with water indirectly stimulates peristalsis. hMW PEG laxatives are the first recommended treatment for constipation in children. They have been shown to be more effective than lactulose (the sweet, sticky syrup laxative), stimulant laxatives (Senna, pico sulphate), milk of magnesia (white liquid, sometimes tastes milky) and probiotics (Ratanamongkol et al., 2009; Chmielewska and Szajewska, 2010; Coccorullo et al., 2010). There are various reasons which make the choice of PEG without ancillary salts the best choice in the options for treatment of occasional and chronic constipation and for resolution of faecal impaction. Firstly, clinical literature shows that hMW PEG is effective, with the advantage of being well tolerated, without side effects or specific contraindications. A study in 2003 looked specifically at the clinical and biochemical safety profile of hMW PEG 3350 therapy in children with chronic constipation. It measured the blood levels and overall health of 83 children over 3 months and found that hMW PEG therapy is safe and is well accepted by children with chronic constipation with and without encopresis (Pelham et al., 2008). Similar results have been reported elsewhere (Pashankar et al., 2003), including infants (Gremse et al., 2002).

## 3 Pharmacokinetic of hMW PEGs

### 3.1 Absorption

Several human studies reported on the bioavailability and absorption kinetics of PEGs (Shaffer and Critchfield, 1950; Delahunty and Hollander, 1986; DiPiro et al., 1986; Schiller et al., 1997; Almer et al., 1993; Parlesak et al., 1994). While absorption of lMW PEGs have been reported, hMW PEGs (i.e., PEG 3350 and 4000) exhibited a negligible absorption in humans (DiPiro et al., 1986; Parlesak et al., 1994; Schiller et al., 1997; Pelham et al., 2008). For lMW polymers, urinary recovery, indicative of systemic absorption of the polymers, accounted for approximately 20%–36% of the administered dose in animals (Shaffer and Critchfield, 1950) and as much as 60% in healthy humans (Shaffer and Critchfield, 1947; Chadwick et al., 1977; Delahunty and Hollander, 1986; Oliva et al., 1994; Parlesak et al., 1994; Eaton et al., 1995). This evidence has been correlated with the capability of lMW PEGs to be absorbed by solvent drag mechanism by using aquaporins (Krugliak et al., 1989; Kim, 1996). They are ubiquitous membrane proteins that, in osmotic processes, transport not only water but also some solutes (Gomes et al., 2009).

Unlike lMW PEGs (i.e., 400, 600), hMW PEG passes through the gastrointestinal tract without being absorbed into the bloodstream (van Wijck et al., 2012; Schiller et al., 1997). Indeed, it is a polymer made up of long chains and therefore cannot pass through the intestinal wall; the human gut does not have any specific transporters for hMW PEGs. This avoids systemic consequences and ensures that its effect manifests locally in the intestine. The usefulness of hMW PEG as an infusible marker for measurement of intestinal absorption was confirmed by total intestinal perfusion studies in humans using solutions containing 1 g/l PEG 3350 (Schiller et al., 1997). Effluent recovery of PEG 3350 was measured at 99% ± 4% and 109% ± 2%, confirming it is essentially not absorbed in the human intestinal tract. In the lumen, due to their highly hydrophilicity, hMW PEGs attract and retain water in the stool but also prevents its absorption through the intestinal mucosa. Moreover, it is a chemically inert polymer and consequently does not react with other substances or enzymes in the gastrointestinal tract, avoiding its degradation and therefore its potential absorption.

hMW PEGs might be absorbed in small quantities by passive diffusion in presence of osmotic pressure that could occur following their administration as diluted solutions in water. However, their strong thermodynamic interaction with water (Ohki et al., 2007), approximately three water molecules are bound to each ethylene glycol repeat unit in the polymer, increases significantly their steric hindrance and, therefore, block their paracellular transport through the gastrointestinal epithelium.

Although hMW PEGs lack dedicated transport mechanisms and are intrinsically excluded from transcellular and paracellular uptake under physiological conditions, it is important to acknowledge that intestinal permeability is not static. Several pathological states can transiently alter barrier integrity and, in principle, modulate the fate of macromolecules. Conditions characterized by mucus depletion or altered mucin composition—such as active ulcerative colitis, Crohn’s disease, or severe infectious enteritis—may reduce the steric and electrostatic filtering normally provided by the mucus layer (Pelaseyed et al., 2014), although available evidence indicates that PEGs above 3–4 kDa still experience substantial size-dependent exclusion (Camilleri et al., 2012). Similarly, epithelial injury, erosions, or increased epithelial turnover, as observed in chemotherapy-induced mucositis or celiac disease during active inflammation, can transiently increase paracellular leakiness; however, even in these settings, the upper size cutoff for permeation remains far below the hydrodynamic radius of PEG 3350/4,000 (Turner, 2009). The molecular architecture of tight junctions imposes a stringent steric limit—typically below 1 nm—that effectively excludes polymers of the size of PEG 3350/4,000, even under conditions of junctional dysregulation (Shen et al., 2011). Finally, compromised barrier states such as short-bowel syndrome, severe malnutrition, or critical illness are associated with increased permeability to small solutes and peptides, but no clinical or experimental data support meaningful absorption of high-molecular-weight PEGs. Taken together, these studies indicate that although pathological conditions can modulate intestinal permeability, the steric and structural constraints imposed by mucus organization and tight junction architecture remain the dominant determinants of macromolecular exclusion, making significant absorption of PEG 3350/4,000 unlikely even in diseased states.

**FIGURE 1:**
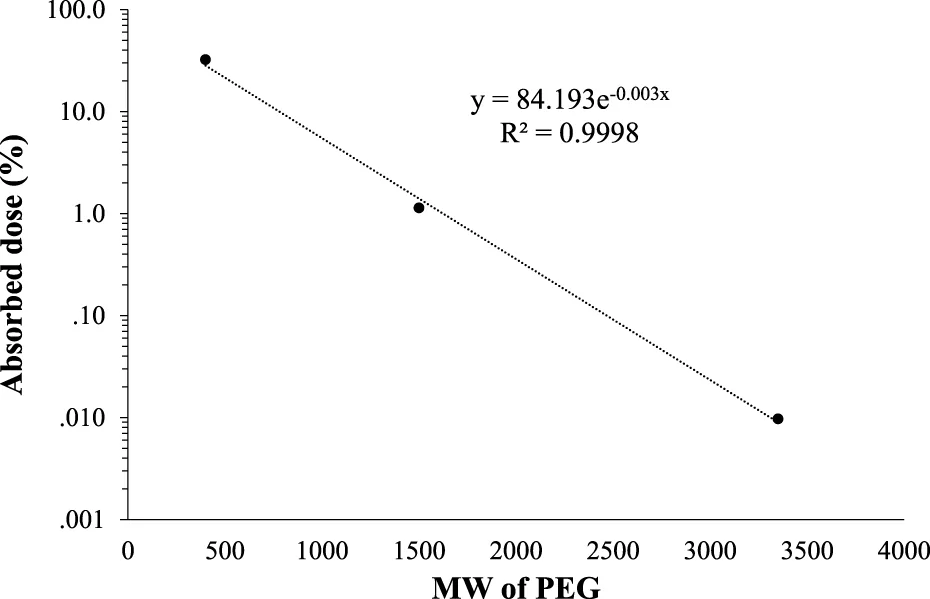
Illustrative relationship between PEG molecular weight and reported urinary recovery based on data provided by van Wijck et al., 2012. The exponential curve is intended only as a visual aid to show the molecular-weight-dependent decrease in PEG absorption and should not be interpreted as a statistically validated predictive model.

Altogether, the previously cited human studies reveal that the degree of absorption following oral administration of PEGs is dependent on the MW of the polymer. Unlike lMW PEGs, a negligible extent of gastrointestinal absorption of several hMW PEG was demonstrated following their administration to rats and to humans. In rats no absorption of the hMW PEGs was noted, whereas it was for lMW PEGs (Shaffer and Critchfield, 1947). On humans, van Wijck and co-workers (van Wijck et al., 2012) suggested the existence of a correlation between the absorbed dose of PEG and its MW (Figure 1). Whereas approximately 32% of PEG 400 was found in urine, only 0.01% was found for PEG 3350. In line with such trend, a negligible permeability of PEG 4000 is expected; for such, PEG 4000 is generally used as no-permeable model drug for checking intestinal epithelial integrity. This is the case of the ICH M9 guideline on biopharmaceutics classification system-based biowaivers, where PEG 4000 is included as “zero-permeability” model drugs for validating Caco-2 cell permeability assay method (EMA, 2020).

As well, the negligible absorption of hMW PEGs in humans is in line with Summaries of Products Characteristics of hMW-PEG-based medicinal products (AIFA, 2026) and with other literature findings on healthy volunteers and patients in which the absorbed fraction is <0.3% of administered doses for PEG 3350 g/mol and <0.08% for PEGs of higher MW (Pelham et al., 2008; Brady et al., 1986). However, safety concerns may arise from the certain degree of variability in the absorbed fractions reported in the literature: e.g., on PEG 3350 ranged from 0.01% (van Wijck et al., 2012) to 0.28% (Pelham et al., 2008).

The integration of historical data on lMW PEGs shows a clear MW-dependent gradient: systemic absorption ranges from very high values (up to 60%) for lMW PEGs, to values below 4% for PEG 2000, down to levels <0.3% for PEG 3350 and <0.08% for PEG >4000 (Table 1). This pattern confirms that the absorption of hMW PEGs is physiologically limited and consistent with available regulatory and clinical data.

**TABLE 1:** Summary table of reported absorption or urinary recovery for PEGs with different molecular weights.

PEG (MW)	Absorption/urinary recovery (%)	Population/Conditions	References
lMW PEG (400 and 600 g/mol)	Up to 60%*	Healthy subjects	Shaffer and Critchfield (1950); Chadwick et al. (1977); Delahunty and Hollander (1986); Oliva et al. (1994); Parlesak et al. (1994); Eaton et al. (1995)
3350 g/mol***	0.01%**	Healthy subjects	van Wijck et al. (2012)
3350 g/mol***	0.28%**	Healthy subjects	Pelham et al. (2008)
3350 g/mol***	<0.3%	Healthy subjects and patients	AIFA (2026) (summaries of products characteristics)
>4000 g/mol***	<0.08%**	Healthy subjects	Brady et al. (1986)
hMW PEG (3350 and 4000 g/mol)	Negligible absorption	Regulatory data and literature	AIFA (2026) (summaries of product characteristics)

* PEG urinary recovery.

** PEG absorption after oral administration.

*** Molecular weight of administered PEGs.

The main problem limiting these studies regards the analytical methods used to detect and separate high from low molecular weight PEG fractions which, as reported in literature and in USP monograph, are always present. Indeed, it is noteworthy that methods used for the quantification of PEG in blood are subject to relatively high analytical variability and are not suitable for distinguishing the molecular weights of the different PEG species.

For example, Pelham et al. reported a relative error of 15% for the methods used for quantifying PEG in serum; moreover, their methodologies are not enough sensitive and accurate for complaining with USP standards on the determination of PEGs’ MW distribution (USP, 2026). PEG characterization poses several challenges due to its unique properties, as the lack of chromophores makes common direct detection challenging in techniques like UV–vis spectrometry. Moreover, PEG, especially lMW variants, often displays weak retention on standard C18 HPLC columns due to its high hydrophilicity and lack of a strong hydrophobic moiety for interaction with the stationary phase. For efficient analysis, it is critical to select stationary phases that can accommodate the polar nature of PEG, often requiring gradient elution to manage the broad range of molecular weight. PEG does not have a high affinity for some standard HPLC columns; so, the choice of mobile phase and column is crucial, and the polydispersity of hMW PEGs can make it difficult to accurately characterize each individual oligomer. The use of standards with known molecular weights becomes crucial for a correct separation and identification of fractions with different molecular weights. Without standards at different molecular weights, it is still possible to characterize the molecular weight distribution of hMW PEGs, but reliability will depend primarily on mass spectrometry rather than HPLC retention. The analysis by electrospray ionization MS (ESI-MS) becomes more challenging due to the formation of multiple charge states with different types of charges (protons, metal ions, ammonium ions) and the presence of unreacted PEG and PEG chains from degraded conjugates, which results in spectra containing several partially overlapping oligomeric distributions (Bento et al., 2024). Therefore, mass spectrometry must be optimized to avoid the formation of ion clusters, which could complicate the interpretation of the data. Liquid chromatography-tandem mass spectrometry (LC-MS/MS) has the advantages of offering high sensitivity and specificity, making it the technique of choice for pharmacokinetic and biodistribution studies in complex biological matrices (plasma and tissues). However, since PEG generates multiple charges, it is difficult to scan all precursor/product pairs. Moreover, absolute quantification of hMW polymers is challenging without accurate standardization (Zhou et al., 2017). From this point of view, size exclusion chromatography coupled with multi-angle light scattering (SEC-MALS) does not require PEG standards with identical architectures to the sample and it is ideal for determining molecular weight distribution, degree of modification, and the presence of aggregates. However, it has lower sensitivity than mass spectrometry, and it is less suitable for quantifying trace amounts of PEG in complex formulations or biological fluids (Wang et al., 2024). The high-resolution mass spectrometry (HRMS) excels in structural characterization. It allows for the identification and deconvolution of isoforms, protein conjugates and impurities, providing detailed information on individual oligomers. However, due to the polydisperse nature of PEG, data processing is computationally intensive. It often requires coupling with fragmentation techniques such as MS^ALL^ to avoid false positives/negatives during screening (Wang et al., 2024). Therefore, a comparison of the above techniques leads us to conclude that for biological samples (pharmacokinetics), LC-MS/MS dominates due to its ability to reduce background noise. For quality control in manufacturing processes, SEC-MALS is preferred for determining absolute molecular weight. On the contrary, HRMS allows observation of the actual oligomeric distribution and chemical changes at the level of individual repeating units. Regarding sensitivity, LC-MS/MS with QQQ remains the gold standard for quantification limits in the ng/mL range *in vivo*.

Such aspects were not investigated in the literature studies previously cited (Pelham et al., 2008; DiPiro et al., 1986): neither the PEG polydispersity nor PEG fraction absorbed were investigated. Therefore, according to our opinion, it is not possible to assert unequivocally that the absorption detected in these two studies was attributable to PEG 3350; far more reasonably, it could be attributed to lMW impurities in PEG 3350 preparations.

In parallel, it is worth considering that, as other synthetic polymers produced via polymerization, PEGs are typically polymers with a distribution of molecular weights. Therefore, also in hMW PEGs, it is truthful that lMW PEGs (like ∼400 Da) can be present as a small, residual fraction. As a matter of fact, the United States Pharmacopeia (USP) provides guidelines for the analysis of PEG 3350, which include chromatographic methods to assess its purity and molecular weight distribution (USP, 2026). According to USP acceptance criteria, the value of apparent weight-average molecular weight is 3015–3685 g/mol and polydispersity is 90%–110% of the value stated on the label or within the range indicated on the label. As well, an impurity level of not more than 3% is deemed acceptable by the USP for qualifying PEG 3350 as pharmaceutical grade. This means that a minimal presence of fractions with lMW PEGs (e.g., 400 Da) is accepted for hMW PEGs, from a regulatory point of view. Consequently, it cannot be ruled out that the negligible absorbed fraction observed in above-mentioned articles may be attributable to lMW PEG impurities present in the batches of hMW PEGs used in the studies. This hypothesis is indirectly confirmed by experimental results on animal models provided by Winne and Gorig (Winne and Gorig, 1982). They showed that the apparent absorbed fraction of ^14^C-PEG 4000 in rat consisted mainly of impurities of lower molecular that are present in the commercial batches of ^14^C-PEG 4000.

### 3.2 Transport and distribution

Despite the very small absorbed extent of lMW PEGs into the body, no evidence of accumulation in any specific organ is available in the literature (Brady et al., 1986; Pelham et al., 2008).

### 3.3 Metabolism and excretion

PEGs are recovered unmodified, this connoting the absence of metabolic interactions. When given intravenously, they are eliminated mainly through the kidneys, especially for MWs below 20000 Da, essentially unchanged and unmetabolized (Carpenter et al., 1971). On the contrary, when given orally, the pharmacokinetics of hMW PEGs are characterized by rapid fecal elimination (Pelham et al., 2008).

## 4 Influence of the excipients

The excipients included in PEG formulations are widely used in food supplements, medical devices and medicinal products and, except for individual intolerance to some components, they are safe and usually well tolerated at the doses included in medical devices. Their primary role is to modulate osmotic balance and palatability rather than to alter the pharmacokinetics of hMW PEGs. Since PEG retains water in the intestinal lumen through osmosis, electrolytes ensure that fluid exchange between the body and the intestine remains neutral (isotonic). Electrolytes such as sodium, potassium, and bicarbonate maintain isotonicity within the intestinal lumen, preventing excessive fluid shifts and ensuring that water retention remains confined to the gut. Salts and flavoring agents can indirectly influence transit time by modifying the total osmotic load, but available evidence indicates that these effects do not increase systemic absorption of PEG. In fact, the bioavailability of hMW PEGs remains negligible regardless of formulation, as their hydrodynamic radius and lack of transport mechanisms preclude epithelial permeation.

Since the therapeutic effect of PEG is merely osmotic (it retains water in the intestinal lumen), its absorption and metabolism profile are independent of the formulation (McGraw, 2016). hMW PEG is almost entirely excreted through the feces. Excipients can play an indirect role in transit speed. If the formulation includes salts, the total osmotic effect increases, accelerating transit. This reduces the residence time of PEG in the colon but does not alter its elimination pathway.

Safety data from clinical and toxicological studies confirm that excipients used in PEG laxatives do not promote mucosal damage or enhance permeability (Pelham et al., 2008; McGraw, 2016). Rather, they contribute to maintaining luminal hydration and patient compliance (Di Palma et al., 2002). Even in formulations containing electrolytes or buffer systems, PEG is recovered unchanged in feces, supporting the conclusion that excipients modulate osmotic activity in the intestinal lumen without affecting absorption or metabolism (Carpenter et al., 1971; McGraw, 2016). Therefore, the overall safety profile of PEG-based laxatives reflects the inert nature of both the polymer and its excipients.

## 5 Conclusion

In conclusion, based on the available pharmacokinetic data, hMW PEGs (≥3000 g/mol) are not absorbed from the gastrointestinal tract and are excreted unchanged in the feces. It is noteworthy that some published studies have reported negligible absorption of PEG 3350 or PEG 4000 (<0.3% of an oral dose); however, this finding might be likely attributable to the presence of low-molecular-weight impurities in PEG 3350 or PEG 4000 preparations. Indeed, it is well established that lMW PEGs can permeate the gastrointestinal mucosa via passive diffusion, but the analytical methodologies used in those studies were unable to discriminate among PEGs of different molecular weights.

Given that PEG 3350 and PEG 4000 are non-absorbable and not metabolized in the gastrointestinal tract, they are used for both in loco diagnostic and therapeutic purposes. In accordance with the current regulatory framework, they may be marketed as medicinal products when intended for use as non-permeant markers in diagnostic formulations to assess gastrointestinal permeability in patients (MDCG, 2024). Conversely, when intended for therapeutic use as gastrointestinal osmotic laxatives, PEG 3350 and PEG 4000 may be classified as Class IIb medical devices in compliance with Regulation (EU) 2017/745 (MDR) (MDCG, 2021). Indeed, they should be qualified as such in line with Rule 21 of Annex VIII of MDR, on their mechanism of action in the stomach and lower gastrointestinal tract, as neither the compounds nor their metabolites are systemically absorbed.
